# Spermatozoal large RNA content is associated with semen characteristics, sociodemographic and lifestyle factors

**DOI:** 10.1371/journal.pone.0216584

**Published:** 2019-05-23

**Authors:** Enrica Bianchi, Kim Boekelheide, Mark Sigman, Joseph M. Braun, Melissa Eliot, Susan J. Hall, Edward Dere, Kathleen Hwang

**Affiliations:** 1 Division of Urology, Rhode Island Hospital, Providence, Rhode Island, United States of America; 2 Department of Pathology and Laboratory Medicine, Brown University, Providence, Rhode Island, United States of America; 3 Department of Epidemiology, School of Public Health, Brown University, Providence, Rhode Island, United States of America; 4 Department of Urology, University of Pittsburgh Medical Center, Pittsburgh, Pennsylvania, United States of America; Zhejiang University School of Medicine Women’s Hospital, CHINA

## Abstract

Semen analysis is one of the standard diagnostic tools currently used to assess male infertility and reproductive toxicity. However, semen analysis has a limited ability to separate fertile from infertile populations. Additional methods to detect impaired fertility are needed. The purpose of the present study was to evaluate how spermatozoal RNA content varies with sociodemographic and behavior/lifestyle factors, and to determine if spermatozoal large and small RNAs discriminate normal from abnormal spermatozoa. Semen specimens were collected from 133 men aged between 18 to 55 years undergoing semen analysis as part of couple infertility evaluation while 10 proven fertile donors were recruited as control group. Semen samples were classified as normal or abnormal according to World Health Organization (WHO) 2010 criteria. Sperm RNAs were extracted after somatic cells were lysed, and the association of large or small RNA content with semen quality and sociodemographic and behavioral/lifestyle factors was evaluated using a generalized additive model and one-way ANOVA. Inverse relationship was observed between large RNA content and sperm parameters such as sperm count, density and motility. Large RNA content per sperm was significantly increased in semen samples showing abnormal number of round cells. Furthermore, sperm motility was inversely associated with spermatozoal small RNA contents. Grouping donors by the number of semen abnormalities, we observed significant increased spermatozoal large and small RNA content in men with more than two semen abnormalities. Alcohol consumption was strongly associated with increased large RNA per sperm concentration after adjustment for age and BMI. Our study demonstrates a strong relationship between spermatozoal large RNA content and poor semen characteristics that may lead to a role in the assessment of male fertility, and may be used as an endpoint for reproductive toxicology risk assessment.

## Introduction

Semen analysis remains the cornerstone of male infertility factor evaluation in subfertile couples and is the first laboratory test a clinician will order to evaluate male reproductive tract function [1–3]. Semen analysis is also routinely used to assess the reproductive toxicity of environmental or therapeutic agents (Environmental Protection Agency. Guidelines for reproductive toxicity assessment) [4]. The lower thresholds for semen parameters established by the WHO in 2010 include 39 million sperm per ejaculate with 40% motility, and 4% normal morphology [2]. While population based reference, ranges are available for comparison, conventional semen analysis is limited in its ability to predict the fertilizing potential of sperm, and does not address the subsequent complex changes that occur in the female reproductive tract [5, 6]. Since semen analysis alone is insufficient to predict fertility for a couple, there is increasing interest in identifying new sperm markers, diagnostic tests and sperm selection strategies that would be more predictive of fertilization potential. These new approaches applied in clinical andrology may allow the development of models to predict effect on fertility of environmental or pharmacological exposures.

Mature spermatozoa have little cytoplasm and a highly condensed chromatin architecture that is enriched in protamines. The presence of ribonucleic acid (RNA) in mature ejaculated sperm has been previously demonstrated as both transcription and translation occur, not in the cytoplasm of mature spermatozoa, but in the mitochondria [7]. Mature spermatozoa contain several types of RNAs accumulated in their nuclei [8–10]. The 55S mitochondrial ribosomes are actively involved in protein translation in spermatozoa while some of the essential components of the 80S cytoplasmic ribosomes such as 28S and 18S rRNAs are not present [11]. It is well known that a set of functional RNAs are delivered into oocytes contributing to early embryo development, which influence the phenotypic traits of the offspring [7, 12, 13]. Therefore, spermatozoa are not just a vehicle that delivers the male genomic contribution to the oocyte. Upon fertilization, the spermatozoon provides a complete, highly structured, and epigenetically marked genome that, together with RNAs and proteins, plays a distinct role in early embryonic development [13, 14].

Development of sperm RNA biomarkers has been hindered by the difficulty of RNA sample preparation from sperm and the heterogeneity of RNA within an individual semen sample [15–19]. In a previous study, we developed a high-quality standardized protocol for isolation of RNA devoid of contaminating somatic cells, debris, and genomic DNA, from both rat and human sperm making the study of sperm RNAs more accessible to both basic biology and clinical laboratories [20]. In the present study, we investigated whether spermatozoal large and small RNA content may be used to discriminate normal from abnormal human sperm and to identify sociodemographic and behavioral/lifestyle stressor effects on male fertility. We measured spermatozoal large and small RNA content in patients presenting to the Brown Urological Clinic for evaluation of male factor infertility while we used as control group proven fertile donors.

## Materials and methods

### Study subjects

Human semen samples were collected between 2013–2015 from a total of 133 male patients aged between 18 to 55 years presenting to the Division of Urology (Providence, RI, USA) for male factor infertility evaluation. Men 18–55 years old (n = 10) who had at least one child and were presenting for a vasectomy were recruited between 2017–2018 for this study as the control group. Exclusion criteria were based on sperm availability. Men who were azoospermic due to testicular failure or obstruction were not studied, as they had no sperm available. All other male patients age 18–55 were considered eligible, regardless of race or ethnicity. Semen samples were collected in sterile conical tubes after 2–5 days of sexual abstinence and allowed to liquefy at 37 °C for 30 minutes before clinical diagnosis according to World Health Organization guidelines [21]. Sperm morphology data were not assessed for this study. The study was approved by the Rhode Island Hospital Institutional Review Board Protocol #403908 and this investigation was conducted according to the principles expressed in the Declaration of Helsinki. Participants involved in this study gave written informed consent to use their semen samples for research. To participate in the study, the patients need to complete a questionnaire regarding weight, height, ethnicity, smoking, alcohol and caffeine consumption, exercise, medical conditions in the last 6 months, and medications taken in the last 3 months.

### RNA isolation

Spermatozoal RNA was extracted at the time of each semen collection following semen analysis as described previously [20]. Briefly, human semen samples were washed with warm sperm wash media (Irvine Scientific, Santa Ana, CA, USA) and somatic cells were lysed using somatic cell lysis buffer (SCLB) made of 0.05% SDS and 0.25% Triton X-100. The sperm cells were then washed with PBS and RNA was isolated using the mirVana miRNA isolation Kit (Life Technologies, Waltham, MA, USA). Sperm RNA samples were split into two fractions, large RNA and small RNA. Sperm RNA quantity and quality were assessed using the NanoDrop Spectrophotometer and Agilent 2100 Bioanalyzer (Agilent Technologies, Santa Clara, CA, USA) an RNA-specific electrophoretic chips. The large and small RNA yield per μL was determined by NanoDrop Spectrophotometer. Data were normalized to take into account differences of total sperm count per ejaculate prior to statistical analysis.

### Statistical analysis

We performed statistical analysis using GraphPad Prism 7 software, SAS and R Studio. We used generalized additive models, a non-parametric regression model, to characterize potential non-linear curvature relations of spermatozoal large and small RNA yields in function of semen quality endpoints [22]. Because large and small sperm RNA values were not normally distributed, we ln-transformed them to approximate normality in our regression models. Then, we used generalized additive models (GAMs) to evaluate the nature of the relation between traditional semen parameters and sperm RNA content. GAMs are a flexible regression model that do not assume a linear relation between the outcome and predictor and can use parametric, semi-parametric, and non-parametric functions to describe the mean outcome (i.e., sperm RNA content) as a function of the predictor (i.e., traditional semen parameters). In addition, the GAM model provides a p-value testing whether there is a departure from linearity, where low p-values indicate statistically significant departures from linearity.

Student’s t-test was used to assess statistical differences in the log-transformed spermatozoal large and small RNA contents between semen samples divided in clinically normal or abnormal according to World Health criteria 2010 criteria.

One-way ANOVA was used to assess RNA contents per sperm as a function of sociodemographic and lifestyle factors. These factors included alcohol intake (none, 1–7 drinks/week, and > 7 drinks/week), caffeine intake (0–0.5 cups/day, > 0.5 to 4 cup/day, >4 cups/day), smoking (never, current, and former), exercise frequency (0 times per week, >0 to 3 times per week, >3 times per week), age (continuous, years), body mass index (continuous, Kg/m^2^), race/ethnicity (non-Hispanic white vs all others). Caffeine totals consumed from coffee, tea (non-herbal) and soda were added together to create a variable for daily consumption. Chocolate was not included in the caffeine intake. We adjusted our analysis of alcohol for age and BMI since they were associated with small RNA content per sperm at an alpha of 0.2. One-way ANOVA was also used to compare large and small RNA content in donors showing normal semen parameters, one abnormal semen parameter, two abnormal semen parameters and more than two abnormal semen parameters with proven fertile donors. Data were presented as mean ± standard error of the mean (SEM). Values were considered to be significant at p-value < 0.05.

## Results

**Fig 1:**
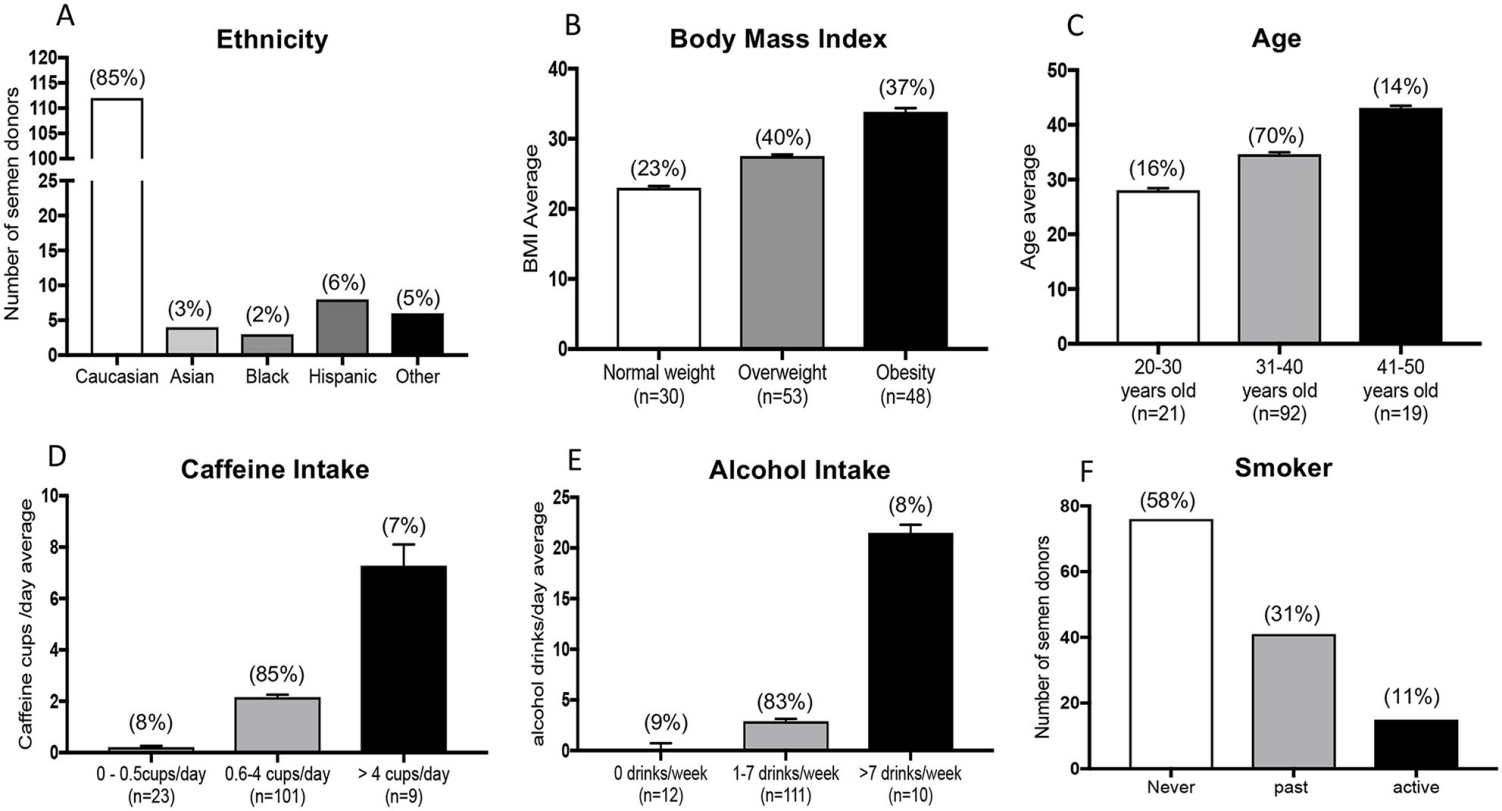
Demographic and lifestyle characteristic of the study semen donor population. A) Ethnicity; B) Body Mass index (BMI): <18.5 underweight, 18.5–24.9 normal weight, 25–29.9 overweight, ≥ 30 obese; C) Age: 20–30 years old, 31–40 years old, 41–50 years old; D) Caffeine intake: 0–0.5 cups/day, 0.6–4 cups/day, > 4 cups/day; E) Alcohol intake: 0 drinks/day, 1–7 drinks/day, > 7 drinks/day; F) Smoke intake: never, past smoker, active smoker.

One-hundred and thirty-three male donors conforming to the inclusion criteria participated in the study; 85% of donors were White, 3% Asian, 2% Black, 6% Hispanic and 5% other ethnicity (Fig 1A). Overall the mean BMI was 28.8 ± 0.4; most participants were overweight (40%) or obese (37%) (Fig 1B). The mean age was 34.8 ± 0.4 years (Fig 1C). On average, participants consumed 2.2 ± 0.2 cups of caffeine a day; 8% didn’t drink any caffeinated beverages (Fig 1D). Most men consumed 1–7 drinks per week (83%) and few consumed none (9%) or >7 drinks per week (8%) (Fig 1E). Finally, 58% of participants never smoked, 31% were former smokers, and 11% were active smokers (Fig 1F).

Specimens were classified as normal or abnormal according to World Health Organization criteria (3). Overall, 92%, 91%, 92%, 84%, and 77% of participants had normal sperm density (> 15 million/ml), total sperm counts (>40 million/ejaculated), sperm motility (>40%), semen viscosity, and absence of agglutination, respectively (Table 1).

**Table 1:** Semen analysis report of semen donor population.

Semen parameters	Mean (± SEM)	Median	5^th^ Percentiles	95^th^ Percentiles	Normal sample according to WHO 2010 criteria (%)
Sperm volume (mL)	3.01 ± 0.12	2.70	1.00	5.50	89
Sperm density (10^6^/mL)	65.23 ± 4.07	53.00	11.60	162.20	92
Sperm count (10^6^/ejac)	188.39 ± 14.24	138.00	26.44	529.40	91
Motility (%)	57.13 ± 1.06	60.00	30.80	73.80	92
Round Cells (10^6^/mL)	0.59 ± 0.07	0.40	0.00	1.84	84
Viscosity	/	/	/	/	69
Sperm agglutination	/	/	/	/	77

Normal semen samples according to WHO 2010 criteria: Sperm volume 1.5–5.5 ml; Sperm density ≥ 15 million/ml; Sperm count ≥ 40 million/ejaculate; Motility ≥ 40%; Round cells < 1 million/ml; Sperm agglutination 0. Total number of semen samples = 133.

**Fig 2:**
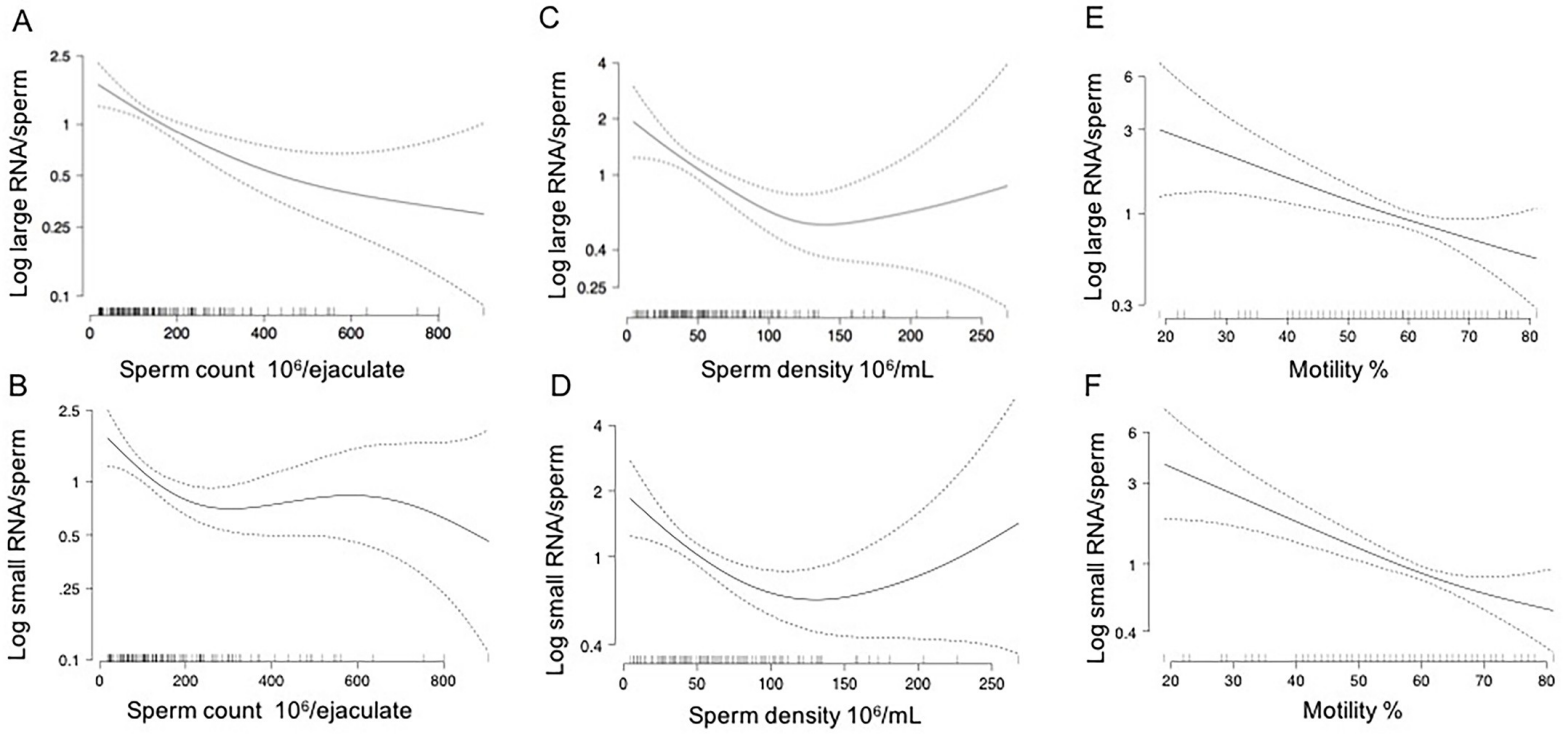
Relation of large and small RNA content per spermatozoon with total sperm count, sperm density and sperm motility modeled with a generalized additive model. A) Large RNA content per sperm was inversely associated with total sperm count (p-value = 0.0003); B) Non-linear curvature relationship between small RNA per sperm content and total sperm count (non-linearity p-value = 0.037); C) Large RNA content per sperm was inversely associated with sperm density (pvalue = 0.0050). D) Non-linear curvature relationship between small RNA per sperm content and sperm density (non-linearity p-value = 0.01). E) Large RNA content per sperm resulted inversely associated to sperm motility (p-value <0.0001); F) Small RNA content per sperm was negatively associated with changes in sperm motility (p-value <0.0001). Data were analyzed using the generalized additive model (GAM). The solid line represents the fitted mean curve of each independent variable; the area between the two dashed lines represents the lower and upper 95% confidence intervals.

Using GAMs, both total sperm count (p-value = 0.0003, Fig 2A) and sperm density (p-value = 0.0050, Fig 2C) were inversely associated with large RNA content per sperm but not small RNA content (Fig 2B and 2D). However, we observed a significant non-linear association between both sperm parameters and small RNA content per sperm using GAMs (non-linearity p-values <0.05; Fig 2B and 2D). Increasing sperm count and density were associated with the declines in small RNA content per sperm up to concentrations of ~200 x 10^6^ sperm and densities up to ~100 x 10^6^ sperm/million, respectively. Total sperm count and sperm densities above these levels were not associated with small RNA content.

**Fig 3:**
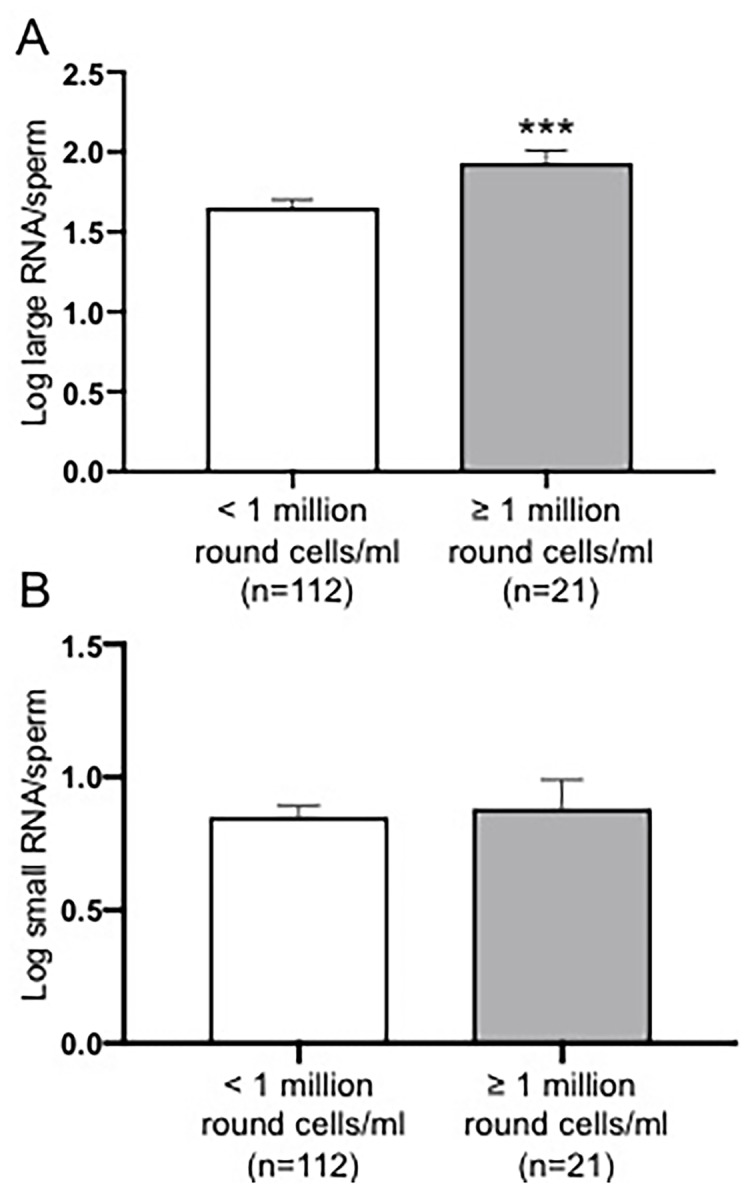
Spermatozoal large and small RNA contents in donors grouped according to the clinical normal or abnormal ranges in the number of round cells. A) Spermatozoal large RNA content was significantly higher in semen samples with 1 million/mL round cells or more compared to the semen samples presenting less than 1 million/mL round cells (p-value = 0.0032); B) No relationship was found between spermatozoal small RNA content and the number of round cells. Data were analyzed by two-tailed Student’s test and expressed as mean ± SEM (** p<0.01).

Sperm motility was inversely, and linearly, associated with both large (p-value <0.0001, Fig 2E) and small (p-value <0.0001, Fig 2F) RNA contents per sperm. Large RNA content per sperm was significantly higher in men with 1 million/mL round cells or more compared to men with less than 1 million/mL round cells (p-value = 0.0003, Fig 3A). No differences were detected in small RNA content per sperm between semen samples showing normal and abnormal number of round cells (Fig 3B).

**Fig 4:**
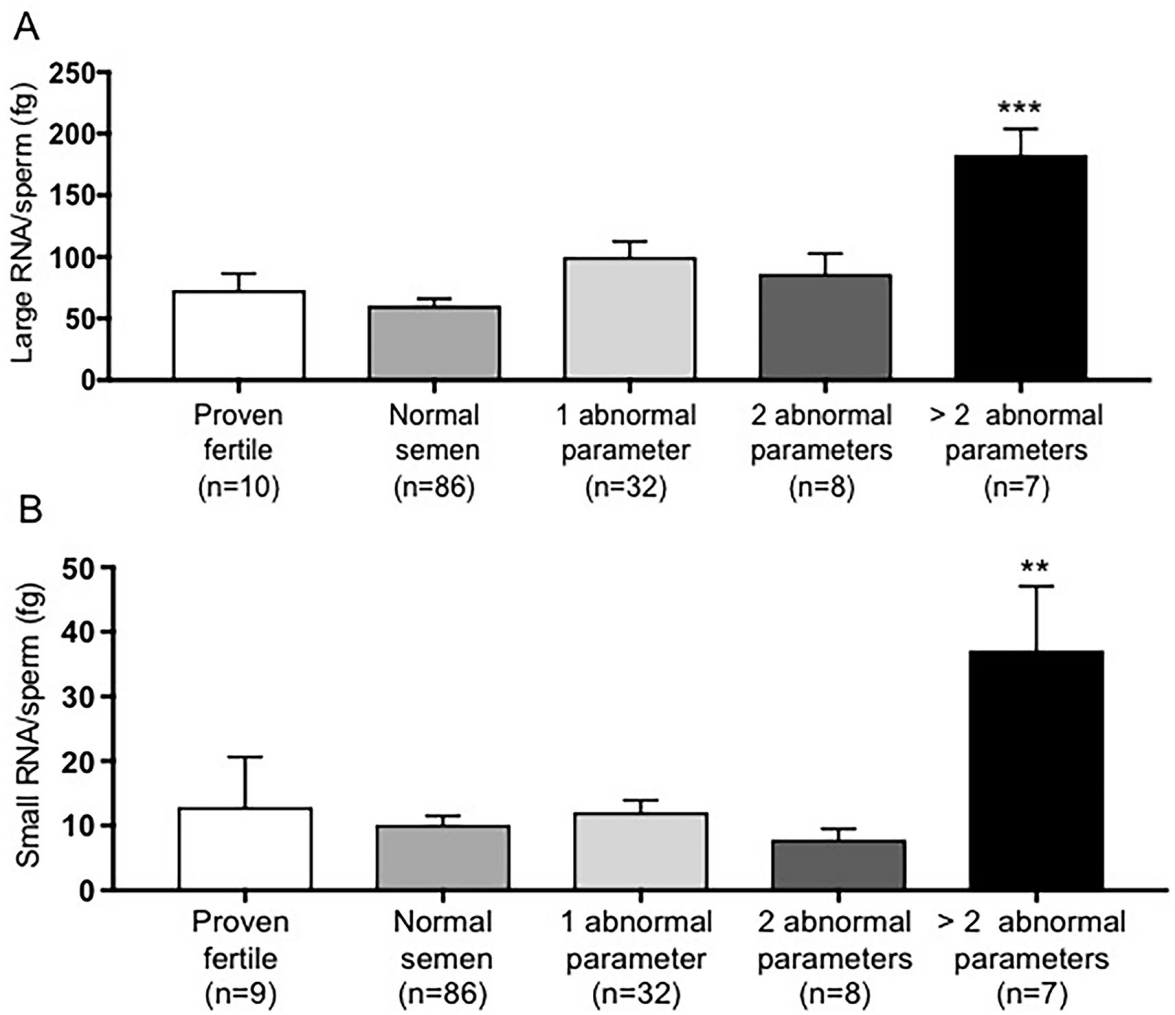
Spermatozoal large and small RNA contents comparison among donors grouped based on the number of semen abnormalities with a comparison to control/proven fertile men. A) Large RNA per sperm content was significantly higher in donors with more than two semen abnormality (p-value = 0.0004) compared to proven fertile donors. B) Small RNA/sperm content was significantly increased in donors with more than two semen abnormal parameters (p-value = 0.0022) compared to proven fertile participants. Data were analyzed by one-way ANOVA followed by Dunnett multiple comparisons and expressed as mean ± SEM (** p<0.01, *** p<0.001).

Large and small RNA content per sperm was measured in donors grouped by the number of semen abnormalities and compared to proven fertile donors. Spermatozoal large and small RNA contents were significantly higher in donors showing more than two conventional abnormal semen quality parameters compared to a control group (p-value = 0.0004, p-value = 0.0022, Fig 4A). Furthermore, Small RNA content per sperm was positively associated with age and inversely associated with BMI, but p-values did not reach conventional levels of statistical significance (Table 2).

**Table 2:** Unadjusted percent difference in large and small RNA according to sociodemographic and lifestyle variables.

		Large RNA		Small RNA	
Variable	N	% Difference (95% CI)	p-value	% Difference (95% CI)	p-value
**Age (years)**^a^	133	2 (-2, 6)	0.3506	3 (-1, 7)	0.125
**BMI (kg/m^2^)**^b^	131	0 (-4, 4)	0.9421	-4 (-8, 0)	0.0615
Ethnicity					
**White**	112	Ref		Ref	
**Non-White**	21	-12 (-49, 52)	0.6383	0 (-42, 74)	0.9887
**Smoking Status**					
**Never**	76	Ref		Ref	
**Former**	42	-22 (-50, 22)	0.2773	-6 (-40, 46)	0.7744
**Current**	15	3 (-46, 97)	0.9356	-14 (-55, 65)	0.6489
Alcohol Consumption					
**None**	12	Ref		Ref	
**1-7/week**	111	107 (4, 311)	0.0387	39 (-30, 176)	0.353
**7+/week**	10	212 (18, 722)	0.0216	149 (-6, 557)	0.0663
Exercise Frequency					
**0/week**	25	Ref		Ref	
**0-3/week**	72	16 (-32, 100)	0.5783	-7 (-45, 59)	0.8002
**3+/week**	35	7 (-42, 96)	0.8356	-3 (-47, 78)	0.9304
**Statin Use**					
**No**	126	Ref		Ref	
**Yes**	7	-41 (-76, 45)	0.2512	-43 (-77, 38)	0.213
Caffeine Consumption					
**0/day**	11	Ref		Ref	
**1/day**	31	41 (-37, 213)	0.4004	25 (-44, 178)	0.5896
**2/day**	52	-16 (-60, 80)	0.6623	-5 (-55, 103)	0.8942
**2+/day**	39	-13 (-60, 89)	0.7241	-17 (-62, 82)	0.647

^a^-Difference in sperm RNA per year increase in age;

^b^-Difference in sperm RNA per kg/m^2^ increase in BMI.

**Fig 5:**
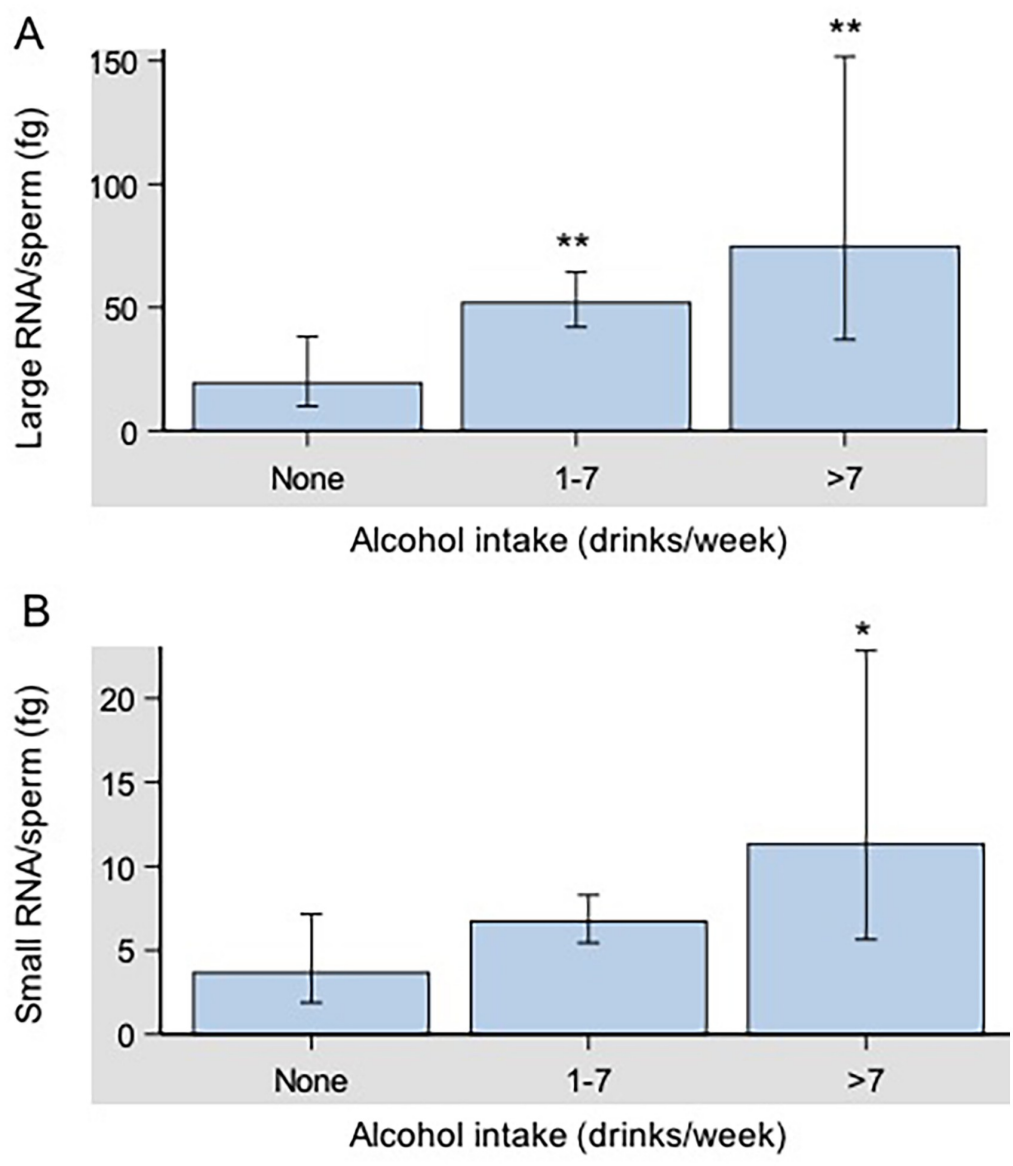
Geometric mean large and small RNA contents per spermatozoon according to self-reported alcohol consumption. A) Large RNA/sperm content was significantly higher in semen samples of donors with moderate (1–7 drinks/day, p-value = 0.0065) and higher (>7 drinks/day, p-value = 0.0067) alcohol intake compared to the non-drinkers (0 drinks/day). A) Small RNA/sperm content was significantly higher in human semen samples of donors with high alcohol intake (p-value = 0.022) compared to the non-drinkers. No differences were detected in small RNA/sperm content between human semen samples of donors with moderate alcohol intake and the non-drinkers. Data were analyzed by one-way ANOVA followed by Dunnett multiple comparisons and expressed as mean ± SEM (* p<0.05, ** p<0.01).

Finally, alcohol use was associated with large RNA content per sperm (Fig 5A) and marginally with small RNA content per sperm (Fig 5B) after adjustment for age and BMI. Large RNA content per sperm monotonically increased with alcohol consumption (p-value = 0.0133, Fig 5A). A similar, but slightly weaker pattern was observed for small RNA content per sperm (ANOVA p-value = 0.0742) after adjustment for age and BMI. Compared to participants who did not drink, those who drank >7 drinks per week had significantly higher small RNA content per sperm (p-value = 0.022, Fig 5B). Race/ethnicity, caffeine intake, and smoking were not associated with large and small RNA content per sperm (Table 2).

## Discussion

Male infertility evaluation relies upon the traditional semen analysis, which assesses parameters such as semen volume, sperm concentration, motility, morphology and presence of round cells other than mature spermatozoa. However, the observation of normal semen parameters using these WHO criteria does not guarantee male fertility and ability to detect sperm abnormalities [3, 5, 23]. The ongoing improvements in assisted reproductive techniques (ART) have highlighted the importance of sperm evaluation and selection to optimize diagnosis and therapeutic management of infertile couples [24]. To date, methods focused on isolating viable and motile spermatozoa have shown that these parameters are not sufficient to identify the most suitable spermatozoon for fertilization and producing healthy offspring. Furthermore, there is an increasing need to optimize diagnostic and therapeutic management of male infertility and to develop a more efficient approach to identify ‘healthy’ spermatozoa in men showing abnormal semen parameters for better IVF/ICSI outcomes [25].

At fertilization, mature spermatozoa in addition to delivering the paternal genome, provide paternal RNAs and proteins to the zygote, indicating that an evaluation of these sperm components could be used as a non-invasive approach to investigate male factor infertility [26]. Although mature spermatozoa were at first believed to be incapable of transcribing RNA, currently there are no doubts about the transcriptional activity present in the mitochondria of mature spermatozoa [7, 27, 28]. Therefore, spermatozoon carries mRNAs that under certain circumstances can be translated de novo by 55S mitochondrial ribosomes during sperm capacitation leading to successful fertilization [11]. Spermatozoal RNAs, including small RNAs, may also have a role in modulating gene expression influencing phenotype through an epigenetic alteration [29]. Therefore, advances in microarray technologies and RNA-sequencing have enabled global analyses of spermatozoal RNAs contributing to the understanding of RNA complexity and molecular mechanisms of male infertility [30, 31]. These new technologies and the development of a high-quality sperm RNA isolation protocol will allow the development of potential clinical sperm biomarkers for male infertility evaluation.

The present study demonstrates that spermatozoal RNA content may be used to evaluate human semen quality and the impact of lifestyle factors on male fertility. Spermatozoal large RNA content was significantly inversely related to total sperm count and sperm density, indicating that poor semen quality is associated with higher spermatozoal large RNA content. Moreover, we observed non-linear associations of small RNA content per sperm with total sperm count and sperm density, suggesting that changes in these traditional semen parameters in the lower range of clinically normal are associated with changes in sperm small RNA content. Furthermore, both large and small RNA content per sperm were inversely associated with sperm motility (Fig 2).

According to the WHO 2010 recommendations, a normal semen sample should contain <1 million/mL round cells [2]. Round cells observed in semen samples could be either of spermatogenic origin or cells of non-spermatogenic origin such as epithelial cells, neutrophils and lymphocytes [32]. The presence of leukocytes may be associated with an inflammatory reaction of the male genital tract interfering with the fertilization ability of spermatozoa [33–35]. It has been reported that the presence of leukocytes in human semen results in a loss of motility [36]. We observed that changes in the number of round cells in the clinical abnormal range were associated with higher spermatozoal large RNA content (Fig 3). Furthermore, the amount of spermatozoal large and small RNAs were strongly increased in men with more than two semen abnormalities compared to a control group consisting of proven fertile donors with normal semen parameters (Fig 4). Finally, large RNA per sperm was increased in association with higher alcohol consumption while small RNA content was correlated with participants who drank more than 7 drinks per week after adjustment for age and body mass index (Fig 5).

These observations are in agreement with a previous study showing that abnormal spermatozoa, defined according to WHO 2010 criteria, have higher total RNA than normal spermatozoa [37]. However, the same group, a few years later, showed that morphologically normal sperm present higher RNA content than abnormal sperm [38]. These conflicting findings from the same group may be due to the RNA isolation protocol used and somatic cell contamination.

The increased content of RNA per sperm in abnormal semen specimens or semen affected by lifestyle stressors may be explained by mechanisms such as impaired spermatogenesis where the spermatozoon is not fully maturing causing excess of RNA per individual sperm cell. This phenomenon may be due to a defect in the transcriptional pathway, an error during replication, defects during gene transcription, or an inability of spermatozoa to perform efficient translation causing an excess of RNA to accumulate [37, 38].

In conclusion, our findings suggest that large RNA content per spermatozoon has the potential to predict sperm abnormalities, and may be useful as a clinical diagnostic tool to assess sperm quality. Sperm content of large RNAs was a more robust predictor than small RNA content. Therefore, the assessment of sperm large RNA contents could be useful in screening sperm for successful ARTs, discriminating normal from abnormal sperm as a selection strategy to maximize reproductive success. The absence of pregnancy outcomes after semen specimens were collected constitutes a limitation of this study. Based on these findings, additional investigations will be necessary to assess the predictive value of large and small sperm RNA contents for pregnancy and fertility.

## Supporting information

S1 Fig Spermatozoal large and small RNA contents in donors grouped in clinically normal or abnormal according to WHO 2010 criteria. Spermatozoal large RNA and small RNA contents were significantly higher in donors with abnormal total sperm count (A, p = 0.003; B, p = 0.0039), sperm density (B, p = 0.0004; E, p = 0.0003) and motility (C, p< 0.0001; F, p = 0.0009) compared to the clinically normal ones. Data were analyzed by two-tailed Student’s test and expressed as mean ± SEM (** p< 0.01, *** p< 0.001, **** p< 0.0001).
(TIF)
