# Supplementary figures and images for: Spermatozoal large RNA content is associated with semen characteristics, sociodemographic and lifestyle factors
Source: PLoS One. 2019 May 23;14(5):e0216584. doi: 10.1371/journal.pone.0216584 (PMC6532849; doi:10.1371/journal.pone.0216584)

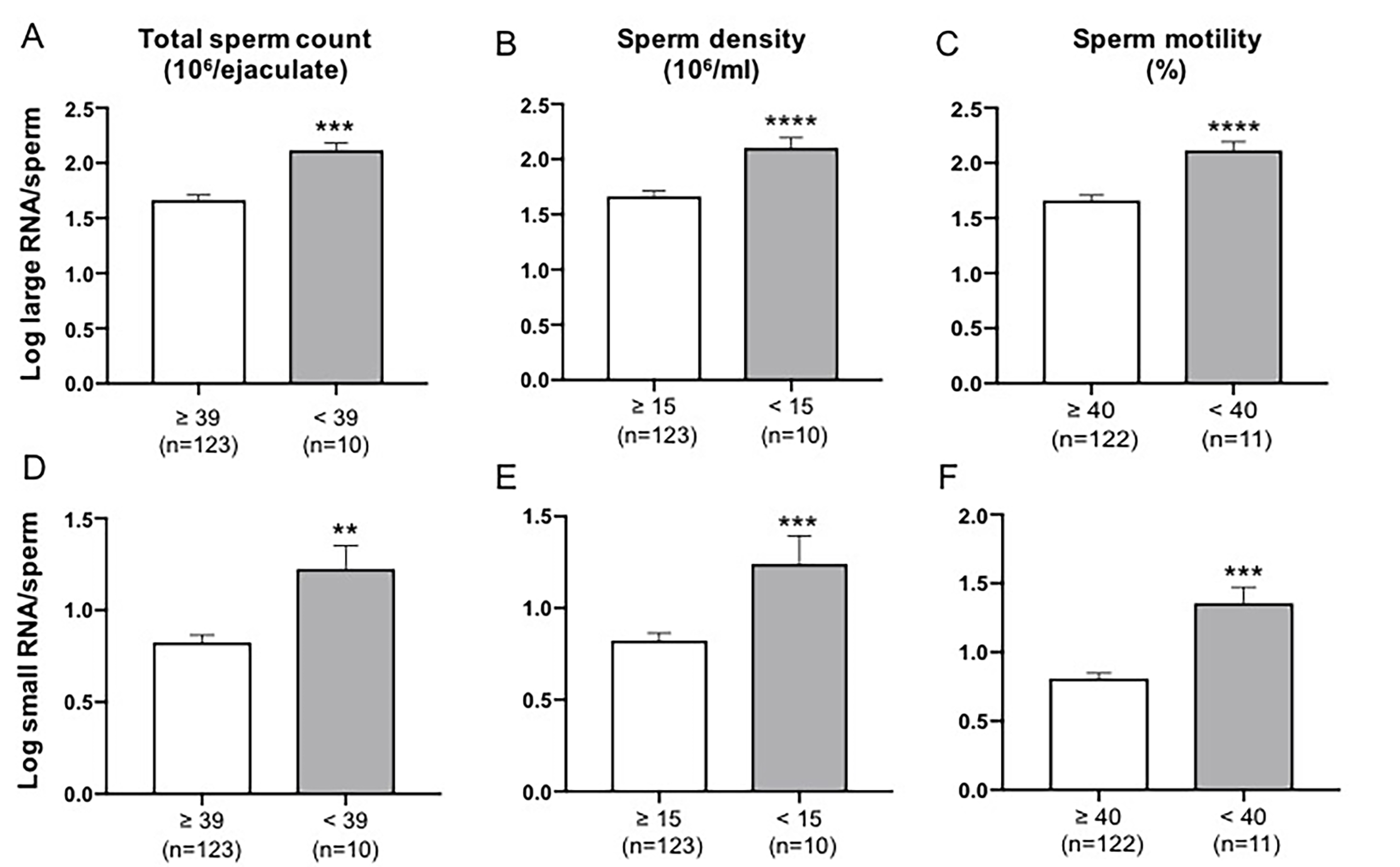

Supplement: S1 Fig — Spermatozoal large RNA and small RNA contents were significantly higher in donors with abnormal total sperm count (A, p = 0.003; B, p = 0.0039), sperm density (B, p = 0.0004; E, p = 0.0003) and motility (C, p< 0.0001; F, p = 0.0009) compared to the clinically normal ones. Data were analyzed by two-tailed Student’s test and expressed as mean ± SEM (** p< 0.01, *** p< 0.001, **** p< 0.0001). (TIF) [file pone.0216584.s001.tif]
